# Deciphering the Immunomodulatory Role of Cyclin-Dependent Kinase 4/6 Inhibitors in the Tumor Microenvironment

**DOI:** 10.3390/ijms24032236

**Published:** 2023-01-23

**Authors:** Pratibha Pandey, Fahad Khan, Tarun Kumar Upadhyay, Amit Baran Sharangi

**Affiliations:** 1 Department of Biotechnology, Noida Institute of Engineering and Technology, 19, Knowledge Park-II, Institutional Area, Greater Noida 201306, India; 2 Department of Biotechnology, Parul Institute of Applied Sciences and Centre of Research for Development, Parul University, Vadodara 391760, India; 3 Department of Plantation Spices Medicinal and Aromatic Crops, Bidhan Chandra Krishi Viswavidyalaya, Mohanpur 741252, India

**Keywords:** cancer therapy, cell cycle, CDK4, CDK6, immunotherapy, tumor microenvironment

## Abstract

Cancer is characterized by persistent cell proliferation driven by aberrant cell cycle regulation and stimulation of cyclin-dependent kinases (CDKs). A very intriguing and potential approach for the development of antitumor medicines is the suppression of CDKs that lead to induction of apoptosis and cell cycle arrest. The shift of the cell cycle from the G0/G1 phase to the S phase, which is characterized by active transcription and synthesis, depends on the development of the cyclin D-CDK4/6 complex. A precise balance between anticancer activity and general toxicity is demonstrated by CDK inhibitors, which can specifically block CDK4/6 and control the cell cycle by reducing the G1 to S phase transition. CDK4/6 inhibitors have recently been reported to exhibit significant cell growth inhibition via modulating the tumour microenvironment in cancerous cells. One significant new understanding is that these inhibitors serve important functions in the interaction among tumour cells and the host immune system in addition to being cytostatic. Herein, we discuss the biological significance of CDK4/6 inhibitors in cancer therapeutics, as well as their biological impact on T cells and other important immune cells. Furthermore, we explore the integration of preclinical findings of these pharmaceuticals’ ability to enhance antitumor immunity.

## 1. Introduction

Cell division is a process used by multicellular organisms to establish and maintain homeostasis of the body. In the cell life cycle, it is crucial to decide whether a cell should multiply or expand. Cell cycle control is a critical element in the timing of this selection. Sustained proliferation due to dysregulated cell cycle control system has been recognized as key characteristics for the cancer development [1]. There are two main phases of the cell cycle: S and M. During the S phase, the maternal cell’s chromosomes are divided into two similar chromatids while these two chromatids are split into each of the daughter cells during the M stage [2]. To appropriately pass the genetic information to the upcoming generation of cells is the objective of cell cycle progression. It is important to control when these two phases occur because, if the M stage starts before chromosome duplication is finished, at least one of the daughter cells will not have all of the genetic information [3]. The purpose of checkpoints is to halt the cell cycle from moving forward before the previous stage has finished. Checkpoints comprise detection systems that recognize phase completion as well as detectors that trigger effector pathways, which can halt the cell cycle progression and subsequently stimulate repair pathways [4]. Checkpoints take account of cellular proliferation, DNA replication, DNA damage, and kinetochore attachment to the mitotic spindle to ensure that cell division occurs with high accuracy [5]. Cyclins, cyclin-dependent kinases (CDKs), cyclin-dependent kinase inhibitors (CKIs), and anaphase-promoting complex/cyclosome (APC/C) are all implicated in checkpoint execution [6]. In order to trigger the effectors, the signaling pathway is activated in accordance with the sensors detecting abnormal cell growth, imperfect replication, DNA damage, and spindle errors. In turn, the effectors cause cell cycle inhibition and turn on the proper repair pathways [7,8]. Heterodimeric protein complexes with kinase activity are the principal cell cycle regulators. The heterodimers are mainly composed of a catalytic subunit (CDK) and a regulatory subunit (cyclin) [9]. The CDKs regulate the timing and beginning of the cell cycle stages by binding to various cyclins. Numerous proteins involved in controlling the cell cycle are phosphorylated by these complexes, which may either activate or inhibit each individual protein [10].

With the advancement of contemporary molecular biology and genetics in the 1980s, a better understanding was obtained of the genetic changes underlying unchecked growth in tumor cells. One of the primary pathogenic symptoms of malignant transformation is the prolonged cell proliferation driven by unchecked cell division [11]. The primary abnormalities linked to malignant transformation include the loss of genes encoding cell cycle inhibitors (such as retinoblastoma 1 (RB1) and cyclin-dependent kinase inhibitor 2A) as well as the increased expression of proteins that direct cell cycle progression like cyclin D1 (CCND1), cyclin-dependent kinase 4 (CDK4), and CDK6. Inhibiting abnormal cell division and proliferation is thus a viable approach in the treatment of cancer. Particularly important in the control of cellular proliferation and division are cyclin-dependent kinases (CDKs). A growing number of malignancies are being treated with CDK inhibition because it stops cell proliferation [12,13] The discovery of “key regulators of the cell cycle” made by Leland H. Hartwell, Paul M. Nurse, and R. Timothy Hunt were recognized with the Nobel Prize in Physiology or Medicine [14,15]. These findings have sparked fresh approaches to the treatment of cancer. Over the past 20 years, a large number of medications that target CDKs have been developed to treat malignancies due to the critical role that CDKs play in the regulation of cell division and growth [16,17]. The first- and second-generation CDK inhibitors were stopped during clinical trials despite positive preclinical results because these nonselective pan-CDK inhibitors had severe toxic effects on healthy cells [18,19]. The third generation of CDK inhibitors, which show preferential specificity for CDK4/6 over some other CDKs, have received regulatory approval from the United States FDA for use in the management of breast cancer [20]. There has been a significant increase in experimental research and patents related to CDK4 research during the past ten years. The study of CDK4/6 has consistently been a topic that has attracted a lot of interest. At addition to the three distinct CDK4/6 inhibitors that have been authorized, 15 more CDK4/6 inhibitors are being tested in various phases of clinical trials as anticancer drugs [21].

In addition to regulating the cell cycle during cell division, these proteins have recently been found to have additional biological activities [22,23]. The cell cycle regulators have some noteworthy non-canonical roles that extend beyond the level of the individual cell. These proteins are critical elements of both tissue-tissue and tissue-environmental interactions in multicellular organisms because they have recently been demonstrated to be critical immune response regulators. Surprisingly, translational results from these ongoing studies have shown that CDK4/6 inhibitors play a number of crucial functions, such as cross talk between tumor cells and the host immune system [24,25,26]. Clonal growth and differentiation, which are necessary for the production of an efficient adaptive immune response, are coupled by the cell-cycle cascade, and as a result, CDK inhibitors may influence the choice between anergy, tolerance, and the stimulation of antitumor immunity.

In this review, we highlight the significant functions of CDK4/6 in the control of cell cycle progression in normal cells and describe the several methods by which deregulation of the CDK4/6 pathway leads to unchecked cancer cell proliferation. Additionally, we focus on new experimental and clinical evidence that CDK4/6 inhibition promotes key facets of antitumor immunity in the tumour microenvironment, tipping the balance in favor of the induction of an effective antitumor immune response.

## 2. Cell Cycle Regulation by CDK4/6 in Normal Cells

**Figure 1:**
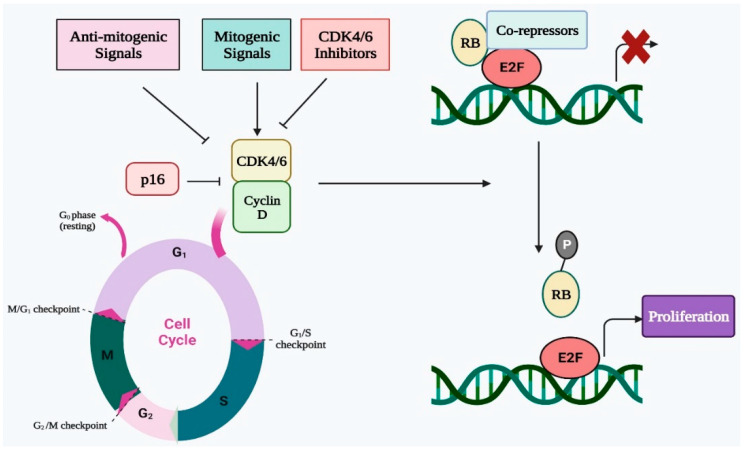
Regulatory role of CDK4/6 in cell cycle progression via E2F-Rb pathway.

Four successive stages make up the cell cycle, which is a highly conserved biological process. These phases are pre-DNA synthesis/G1, S/DNA synthesis, G2/pre-division, and M/cell division. To assure proper progression through the complete cell cycle, many CDKs work with their cyclin partners to govern the transition from one stage to the next. In terms of biochemistry and biology, CDK4 and CDK6 are quite similar to one another, and CDK4/6 can be triggered by D-type cyclins, which are an essential activator of the shift from G1 to S phase [27,28]. In the early G1 stage, the number of D-type cyclins begins to rise in response to proliferative stimuli, whereupon these cyclins interact and bind with CDK4/6 and activate them (Figure 1) [29]. The cyclin D-CDK4/6 complex, which binds with the transactivation domain of the E2F transcription factor family, thereafter phosphorylates the retinoblastoma (RB) protein [30,31]. The E2F transcription factor is released as a result of RB phosphorylation. Additionally, the E2F transcription factor promotes E-type cyclin production before binding to CDK2. The cyclin E-CDK2 complex promotes the G1 to S phase transition while simultaneously accelerating RB phosphorylation and reducing E2F p21 suppression. As a result, CDK4 and CDK6 are essential for the G1 to S phase transition and must be blocked to effectively prevent the G1/S transition [32,33]. The cyclin D-CDK4/6 complex becomes hyperactive as a consequence of a rise in cyclin D concentration or CDK4/6 activity, which speeds up the cell cycle and the transition from the G1 to the S phase. Additionally, an accelerated cell cycle leads to unregulated cell multiplication that causes tumor progression [34]. Thus, CDK4/6 inhibition can result in G1 arrest of the cell cycle, making it a potent and successful approach for cancer therapy. To ensure the effective progression of the cell cycle, cyclin-dependent kinase inhibitors (CKIs) such as INK4 and CIP1/KIP1, regulate CDK activity [35,36]. The CDK4/6 function is restricted by INK4 proteins, which either directly attach to CDK4/6 to inhibit its catalytic property or selectively interrupt the interaction of cyclin D with CDK4/6. In contrast to INK4 proteins, the CIP/KIP proteins engage with all CDKs that are essential for the cell cycle and can either suppress or promote CDK activity based upon the cellular environment [37,38]. Therefore, the function of CKIs is vital for proper CDK function and healthy cell growth, and cancer may develop as a result of the lack of their function.

## 3. Regulation of Immune Response by CDK4/6

A crucial factor in controlling infections is innate immunity, which acts as the primary defense mechanism for the host. Invasive, fatal infections are linked to deficiencies in innate immunity. Auto-inflammatory conditions can result from improper innate immune system generation. Neutrophils, dendritic cells, macrophages, and innate lymphoid cells are the main cellular elements of innate immunity. The later growth of adaptive immune responses is ultimately regulated by the innate immune system; thus, its appropriate performance is necessary for good health. Cell cycle regulators function primarily as innate immune accelerators during the innate immune response [39]. Cell cycle molecules such as CDKs and CKIs directly contribute to the proliferation of innate immune system cells and the maintenance of homeostasis in innate immune responses [40]. Recently, it was discovered that CDKs have a direct, non-cell cycle role in innate immunity. Innate immune cells, such as monocytes, generate type I interferon (IFN) after viral infection to defend the host against viral attacks. The expression of type I IFN (IFN) was suppressed in a monocyte cell line called THP-1 by CDK suppression. It was determined that the activity of CDKs 1, 2, and 4 is necessary for the translation of type I IFN mRNA. IFN-β-mRNA is eliminated from the translational polysome complex in the lack of CDK function directed by a pan-CDK inhibitor such as R547, Dinaciclib, but overall translation is unaffected. This shows that CDK activity is especially necessary for the generation of IFN- β, which in turn triggers immune system activation [41]. There must be additional follow-up research on these observations. Since the majority of this study’s conclusions were based on pan-CDK inhibitors, genetic tests in which one or more CDKs are selectively knocked out need to be carried out to confirm the findings. Pan-CDK inhibitors may immediately stop the cell cycle, which would have an impact on the translation of the IFN mRNA. The mechanism by which CDK kinase activity maintains the IFN-mRNA in the translating polysome complex is still unknown [42]. Furthermore, it is unknown how active CDK/cyclin complexes, which are known to be in the nucleus, may regulate cytosolic translation.

Following an initial reaction to a particular pathogen, the adaptive immune system (acquired immune system), develops immunological memory, which results in an improved response to repeated exposures with the infectious agent. The cells that generate the acquired/adaptive immune response are known as lymphocytes. The two main categories of adaptive immunity are antibody and cell-mediated immune responses, which are produced by B and T-lymphocytes, respectively. Genetic studies revealed that particular cell cycle regulators are necessary for the number of adaptive immune cells, as with innate immune cells. Thymocyte counts must be maintained by cyclin D3-CDK6 activity. Mice lacking either CDK6 or cyclin D3 displayed a marked decline in thymocyte counts [43,44]. Additionally, cell cycle proteins indirectly regulate lymphocyte functioning during T cell activation. To create effector cytokines in reaction to secondary exposure, T cells must undergo extra cell cycle rounds during the main response [45,46]. D cyclins connect the growth and activity of B cells, and subsequently B cell clonal growth entails quick proliferation to produce a germinal core (GC). B cells go through class switching, clonal proliferation, and selection within the GC to produce a high-affinity humoral antibody.

In both mice and human dendritic cells, CDK4 is able to phosphorylate the protein kinase mitogen-activated protein kinase 8 (MAPK8; also known as JNK), which triggers the release of IL-6 and IL-12 in response to transcriptional processes that are dependent on AP-1 [47]. Furthermore, because neutrophils are terminally differentiated cells, CDK4 and CDK6 have a role in the capability of neutrophils to react against microbial infections by evading neutrophil extracellular traps, a function that is inversely controlled by the broad-spectrum CDK inhibitor p21CIP1 [48]. These results demonstrate the critical involvement of CDKs in immune system function and development by defining the needs for various CDKs based on cell type and developmental status, as well as a wide range of cell cycle independent functions.

## 4. Dysregulation of CDK4/6 Activity in Cancer

Mutations and deregulation of many cell cycle regulators, including CDKs, cyclins, CKI, CAK, CDK substrates, and checkpoints, have often been detected in malignant cells [49]. The fact that CDK4/6 expression levels are substantially elevated in several cancers is well known [50,51,52,53]. Through both direct and indirect phosphorylation of RB (via inducing CDK2), overexpressed CDK4/6 promote G1/S conversion and promote carcinogenesis. Along with overexpression, CDK4/6 upregulation is a common occurrence in various malignancies (melanoma, leukemia, lymphoma, glioma, sarcoma, etc.) with distinct tissue selectivity for CDK4/6. Compared to its homolog, CDK4 favors mesenchymal tissues (including leukemias and sarcomas), and epithelial tumours (i.e., various malignancies), and some sarcomas tend to express more CDK4 [54]. The majority of human malignancies contain wild-type RB [55], and inhibiting hyperactivated CDK4/6 in these cells can stop the cell cycle during G1 stage. Even in RB negative cancers, CDK4/6 inhibitors work by inhibiting cell division or causing apoptosis without involving RB [56]. Targeting CDK4/6 can also prevent their cell cycle-independent tumor-promoting activities. The modulation of inflammatory cytokine signaling by CDK4 in breast cancer has been revealed using transcriptomic analysis [57]. CDK6 can activate stem cells, promote angiogenesis, the immune system, and other processes [58]. In addition, a variety of oncogenes of the signaling pathway promote cell growth by turning on the CDK4/6-RB-E2F pathway. A few examples of these signaling cascades are PI3K/Akt/mTOR, JAK/STAT, RAS/RAF/MEK/ERK, BTK/NF-kB, and Wnt pathways [59,60,61]. Additionally, perturbations in tumour suppressors, such as p53, can trigger the activation of the CDK4/6-RBE2F pathway by relieving the repression of p21CIP1. Thus, CDK4/6 acts as a node in the pathways of carcinogenesis. Despite being optional in normal cells, CDK4/6 have been proven to be essential for the growth of tumour cells in knockout experiments. As a result, it is safe to kill the opposition without endangering allied forces [62]. Together, these characteristics make CDK4/6 attractive and secure targets for anticancer treatment.

## 5. CDK4/6 Inhibitors as Immunomodulators in Cancer Cells

**Figure 2:**
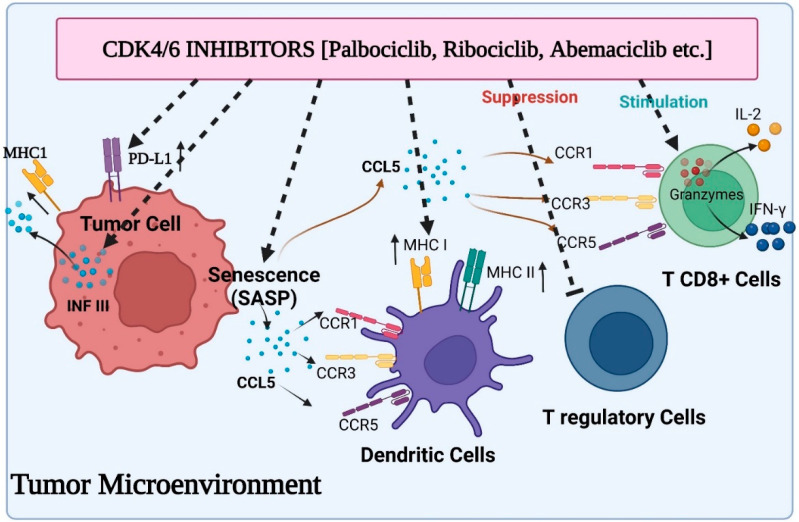
Various aspects of immunomodulation via CDK4/6 inhibitors on the tumor microenvironment: induced activity of antigen presentation and PD-L1 expression on tumor cells, suppressed activity of Treg cells, enhanced cytokine secretion from CD8+ T cells and stimulated function of antigen presentation by dendritic cells.

Recent research has shown that CDK4/6 exhibit substantial cell cycle independent activity in tumour immunosurveillance, and CDK4/6 inhibitors may control the immune system response to tumour cells. First, newly available information suggests that CDK4/6 inhibitors may have an immediate immunomodulatory effect on tumour cells (Figure 2). Abemaciclib treatment in this situation has been associated with the overexpression of multiple antigen processing and antigen presentation machinery elements, including MHC class I molecules (specifically, HLA-A, HLA-B, and HLA-C in human systems, and H2-D1, H2-K1, and B2M in mice systems). OVA-expressing mouse tumour cells become more susceptible to OVA-specific OT-I T cells when exposed to abemaciclib in vitro, and the therapeutic efficacy of abemaciclib in vivo in syngeneic immune-competent animal models is reversed by CD8+ T cell depletion, supporting the idea that MHC class I overexpression plays a mechanistic role in the therapeutic effects of abemaciclib [63,64]. In addition, T cells that invade abemaciclib-treated breast cancer have lower amounts of exhaustion biomarkers such as PD1 and cytotoxic T lymphocyte-associated protein 4 (CTLA4). Moreover, the microenvironment of mice colorectal cancers administered with abemaciclib is characterized by elevated expression of transcripts encoding T cell activation signals such as IFN-γ and granzyme B (GZMB), as well as co-stimulatory receptors such as TNF receptor superfamily member 9 (TNFRSF9).

Following a combination of the PI3K inhibitor BYL719 plus ribociclib or palbociclib, similar outcomes were seen in TNBC models [65]. Researcher revealed that immune-associated pathways were predominant in cancerous cells after receiving therapy with a medication cocktail by conducting a gene set enrichment experiment. Specifically, the elevation of genes associated with antigen presentation and genes coding for cytokines was noticed, suggesting the immunomodulatory impact of these therapies. In contrast to this, new research has shown that CDK4/6 inhibition may support tumour immune evasion in a number of tumour types through the increase of the immunosuppressive programmed cell death protein 1 ligand (PD-L1) expression on tumour cells. Effector T cell functioning in peripheral tissues is regulated by the PD1/PD-L1 immune checkpoint system [66], and PD-1 interaction with T cells directly reduces TCR-mediated effector functions, serving as a negative immune response modulator. Because immune-checkpoint inhibitors have recently been developed, and their activity may be correlated with PD-L1 expression on tumour cells, they may be able to counteract the detrimental immune-suppressive effect of CDK4/6 inhibitors. Accordingly, Zhang and colleagues found an inverse relationship between CDK4 activity and PD-L1 expression on cancer cells, and showed that palbociclib or ribociclib treatment increased PD-L1 expression in a variety of in vitro and in vivo models, regardless of RB status, denoting that the RB/E2F axis was not responsible for this in these models. In fact, the researchers concluded that the Cullin 3-based E3 ligase, which coupled with PD-L1 via the adaptor protein specle-type POZ (SPOP), modulated the level of PD-L1 protein. In particular, CDK4/Cyclin D-mediated phosphorylation and alternate interaction with the proteins 14-3-3γ or FZR1 were responsible for controlling SPOP stability. FZR1 destroyed SPOP, which was no longer phosphorylated in the presence of CDK4/6 inhibitors, boosting the expression of PD-L1 [67]. Palbociclib and an anti-PD-1 antibody treatment significantly prolonged overall survival compared to single medication treatments in mouse models of cancer, which supports the idea that immune-checkpoint antagonists and CDK4/6 inhibitors should be combined to create a more potent anticancer medicine. The enhanced production of PD-L1 by CDK4/6 inhibitors has now been linked to the stimulation of the transcription factor NF-kB in RB-proficient cell lines from various tumour types, as well as an increase in the protein’s durability. It is well known that the NF-kB protein p65 controls the expression of the PD-L1 gene [68]. The interaction of this protein with hyperphosphorylated RB protein can limit the transcriptional activity of RB protein, which in turn inhibits the expression of the PD-L1 gene. As a result, RB loss and CDK4/6 inhibitors both cause the up-regulation of PD-L1 by encouraging the release of active p65 protein. Recently, it was observed that CDK4/6 activity and PD-L1 expression are correlated in melanoma patients [69]. In animal models of melanoma, the mixture of CDK4/6 inhibitors and anti-PD-1 antibodies substantially inhibited cancer growth. It is well established now that CDK4/6 inhibition causes SASP in a number of different cell models. Senescent melanoma cells generated by CDK4/6 inhibitors have been shown to generate CCL5 via activation of NF-kB, a member of the released factors [70]. A chemokine called CCL5 interacts with the CCR5, CCR3, and CCR1 receptors located on a myriad of immune cells, including natural killer cells, immature dendritic, and activated T cells [71]. Researchers revealed that cytokines secreted by senescent cells encouraged the infiltration of immune cells into the cancer using PDX tumours taken from melanoma patients. They found that the expression of markers linked to T lymphocytes (CD2, CD3, CD8A and B, CXCR3, CCR5) and cytotoxic immune cells was positively correlated with the secretion of CCL5 (granzymes and FAS ligand).

## 6. Crosstalk between CDK4/6 Inhibitors and Tumor Microenvironment

In a fraction of cancerous cells, cell-cycle arrest and the initiation of senescence result in the stimulation of SASP. This can cause the migration of innate immune cells, such as natural killer cells, macrophages, and neutrophils, into the tumour microenvironment, where they are stimulated to synergistically attack tumours via phagocytosis and direct cytotoxic death [72,73]. Understanding how tumour cells are driven into reversible quiescence or a more stable senescence remains a major challenge, since preliminary research in a limited group of abemaciclib-sensitive breast cancer cell lines has revealed that genes encoding for the classical SASP cytokines were not increased in the cell lines studied [64]. An RB-proficient transgenic animal model of tumour served as the basis for the initial research, indicating a connection between CDK4/6 regulation and the immune system. In addition to cell stasis, abemaciclib treatment significantly reduced tumour volume and suppressed cell growth. A gene expression study demonstrated that abemaciclib strongly upregulates genes involved in antigen presentation and processing comprising MHC class I molecules, in addition to negatively regulated genes associated with cell cycle, mitosis, and E2F targets. This was verified both in vitro and with xenografts produced from human patients. Surprisingly, tumour cells treated with CDK4/6 inhibitors exhibit a substantial reduction in DNMT1, which lowers DNA methylation activity of endogenous retroviral genes along with immunoregulatory genes. Double-stranded RNA expression causes “viral mimicking”, which facilitates the generation of IFN responses. This approach is further supported by statistics from The Cancer Genome Atlas, which demonstrates that breast tumours with CCND1 amplification concomitantly increase CDK4/6 activity, and exhibit much lower expression of the MHC class I components HLAA, HLA-B, and HLA-C versus tumours without amplification [64,74,75].

Additionally, Deng and coworkers reported that CDK4/6 inhibitors potently increased IL2 levels when they utilized a small-molecule screen to find targets that improved T-cell activity in the presence of PD-1 interaction. They demonstrated through the use of siRNAs that CDK6, not CDK4, was responsible for the increased IL2 release, suggesting CDK6’s predominate role in immune cell function. After careful analysis of the underlying mechanisms behind this impact, it was shown that CDK6 was a crucial upstream regulator of NFAT proteins, which are essential for controlling T-cell activity and performance. Greater nuclear NFAT expression and enhanced transcriptional activity driven by CDK4/6 inhibition altered the cytokine setting in the tumour microenvironment and promoted the activation of effector T cells [24,76]. Three immunosuppressive myeloid cell cytokines-IL6, IL10, and IL23-were substantially downregulated, whereas the Th1 chemokines CXCL9 and CXCL10 that regulate the movement of effector cells to tumour locations were elevated [77].

## 7. Regulatory Role of CDK4/6 Inhibitors on PD-L1 and Other ICIs

In a variety of human cancer cell lines, the level of PD-L1 protein varies as the cell cycle progresses, increasing in M and early G1 and drastically decreasing in later phases. This is closely controlled by the phosphorylation of the speckle-type POZ protein by Cyclin D-CDK4, which is a major element of the Cullin3-SPOP E3 ligase responsible for the proteasomal breakage of PD-L1 [67]. Preclinical studies reported that trilaciclib supplementation to chemotherapy or immune checkpoint inhibitor combinations improved anticancer response and overall survival in small cell lung cancer. Increased effector function and altered T-cell subpopulation composition and growth in the tumour microenvironment are related to this impact. In SCLC patients receiving chemotherapy, transient administration of trilaciclib resulted in preserved and boosted peripheral lymphocyte numbers and improved T-cell activation, indicating that trilaciclib not only preserved but also improved immune function [78]. As a whole, these investigations highlight the intricate relationship between immunology and cell-cycle control. As such, they represent a fascinating new field of study that has the potential to open up a wide range of therapeutic options for the treatment of cancer. The pharmacologic and translational results of the upcoming clinical trials are eagerly anticipated. In several immunocompetent preclinical animal studies, combining CDK4/6 inhibitors and immune checkpoint inhibitors promoted tumour regression [79]. Since the strongest elevation of the antigen-processing machinery at the level of gene expression was observed in CDK4/6-sensitive cell lines, these responses appear to be at least partially tumor-intrinsic [80]. Additionally, there are signs that suggest Cyclin D-CDK4/6 dependence may increase when tumours develop and undergo immune-editing, becoming more immune-refractory. Oh and coworkers examined a malignancy that was highly immune-resistant and observed that synaptonemal complex protein 3 (SCP3) is upregulated in immune-edited cancerous cells, and stimulates the pluripotency transcriptional regulator NANOG through overexpression of the Cyclin D-CDK4/6 axis. Palbociclib and adoptive cytotoxic cell transfer demonstrated significant therapeutic progress in the subject, indicating a niche application for CDK4/6 inhibitors in immunotherapy regimens in the refractory condition [81]. Clinical efficacy of some of the CDK4/6 inhibitors in combination with ICIs is listed in Table 1.

## 8. Conclusions and Future Perspective

There is growing evidence that cell cycle regulators such as CDK4/6 have additional roles in a wide range of biological functions, such as DNA repair, cellular damage, cellular proliferation, metabolic function, and immunological responses. These processes might have a connection with cell division. However, it is unknown why cell cycle regulators perform other functions, particularly systemic ones such as immunomodulation. Among the non-cell cycle roles, the immunomodulatory roles of cell cycle regulators, in particular CDK4/6, are distinctive because they concentrate on cell-cell, cell-environment, or cell-microbes relationship in multicellular animals. Senescence induction, metabolic changes, and immunomodulation are some of the non-canonical outcomes related to CDK4/6 inhibitors that warrant additional investigation to increase the clinical benefit of these medications, perhaps through more efficient combinatorial techniques, and to provide novel treatment prospects even for RB-negative malignancies. Important to note is the interrelationship of all these non-canonical events, which may work in concert to influence how the tumour reacts to CDK4/6 suppression. For instance, SASP may influence the attraction and stimulation of lymphocytes and dendritic cells via secretion of cytokines and chemokines. It would be beneficial to have a stronger insight of the non-canonical CDK4/6 inhibitor effects in order to identify the drawbacks of these therapies, which cannot be projected solely on the basis of canonical actions. To sum up, CDK4/CDK6 inhibitors distinguish themselves as multifunctional immunotherapeutic molecules in disguise, which we consider is crucial for their robust clinical action. We hypothesize that thorough characterization of the molecular pathways by which commercially approved inhibitors affect immune activation would facilitate not only the formulation of new combinatorial combinations with greater antitumor potential, but also the identification of therapeutic interventions to avoid immunoevasion downstream of cell cycle suppression. In this regard, rationally constructed clinical trials and cutting-edge technology for the longitudinal analysis of the cancer microenvironment at single-cell resolution will be crucial.

## Figures and Tables

**Table 1:** List of CDK4/6 inhibitors in combination with immune checkpoint inhibitors/other drugs under consideration or clinically approved in various carcinomas.

Synergistic Drug	Cancer Type	Clinical Trial	Phase	Reference
Etoposide + Carboplatin + Trilaciclib	Small Cell Lung Cancer	NCT02499770	II	[78]
Ribociclib + PDR001–PD-1 inhibitor fulvestrant	HR+ HER2 Negative breast cancer, Epithelial ovarian cancer	NCT03294694	I	[82]
LY3300054-PD-1 Inhibitor + Ramucirumab + Abemaciclib + Merestinib	Solid tumor Microsatellite instability-high (MSI-H) solid tumors Cutaneous melanoma Pancreatic cancer Breast cancer (HRþHER2)	NCT02791334	I	[83]
Abemaciclib + Pembrolizumab	NSCLC HR + HER2- breast cancer	NCT02779751	I	[84]
Palbociclib + Avelumab	Androgen-receptor–positive triple-negative breast cancer	NCT04360941	I	-
Pembrolizumab + Letrozole+ Palbociclib	Metastatic Estrogen Receptor Positive Breast Cancer	NCT02778685	II	[85]
Abemaciclib+ Loperamide + Anastrozole	HER2 Negative Breast Cancer	NCT02441946	II	[86]
Ribociclib + Paclitaxel + Carboplatin	Recurrent Platinum Sensitive Ovarian Cancer	NCT03056833	I	[87]
Abemaciclib + Fulvestrant	Hormone Receptor Positive HER2 Negative Breast Cancer	NCT02107703	III	[88]
Palbociclib + Cetuximab	Metastatic Colorectal Cancer	NCT03446157	II	[89]

## Data Availability

Not applicable.
